# Development of Polymerase Chain Reaction–High-Resolution Melt Assay for Waterborne Pathogens *Legionella pneumophila*, *Vibrio parahaemolyticus*, and *Camplobacter jejuni*

**DOI:** 10.3390/microorganisms12071366

**Published:** 2024-07-03

**Authors:** Shannon M. Carr, Kelly M. Elkins

**Affiliations:** Forensic Science Program, Chemistry Department, Towson University, 8000 York Road, Towson, MD 21252-0001, USA

**Keywords:** waterborne pathogens, *Legionella pneumophila*, *Campylobacter jejuni*, *Vibrio parahaemolyticus*, real-time PCR melt assay

## Abstract

*Legionella pneumophila* is the waterborne pathogen primarily responsible for causing both Pontiac Fever and Legionnaire’s Disease in humans. *L. pneumophila* is transmitted via aerosolized water droplets. The purpose of this study was to design and test primers to allow for rapid polymerase chain reaction (PCR) melt detection and identification of this infectious agent in cases of clinical or emergency response detection. New PCR primers were designed for this species of bacteria; the primer set was purchased from IDT and the target bacterial DNA was purchased from ATCC. The *L. pneumophila* primers targeted the macrophage infectivity potentiator gene (*mip*), which inhibits macrophage phagocytosis. The primers were tested for specificity, repeatability, and sensitivity using PCR–high-resolution melt (HRM) assays. The primer set was found to be specific to the designated bacteria and did not amplify the other twenty-one species from the panel. The *L. pneumophila* assay was able to be multiplexed. The duplex assay consists of primers for *L. pneumophila* and *Vibrio parahaemolyticus*, which are both waterborne pathogens. The triplex assay consists of primers for *L. pneumophila*, *V. parahaemolyticus*, and *Campylobacter jejuni*. The unique melting temperature for the *L. pneumophila* primer assay is 82.84 ± 0.19 °C, the *C. jejuni* assay is 78.10 ± 0.58 °C, and the *V. parahaemolyticus* assay is 86.74 ± 0.65 °C.

## 1. Introduction

The Gram-stain is a widely used method for the initial screening of bacterial pathogens to determine their general type. It has limited utility in cases in which all suspected agents are Gram-negative. As the Gram-stain is used to check for potential pathogens in a bodily secretion sample or from a wound site, a Gram-negative bacterium could be difficult to locate amongst the human cells, which will also stain the same pink as a Gram-negative pathogen, although the human cells are much larger. The bacteria would need to be cultured separately to separate them from the obscuring human cells to better enable their detection. Some bacteria are difficult—if not nearly impossible—to culture, unless very specific conditions are provided. In this study, we focused on the detection and identification of three Gram-negative pathogens: *Legionella pneumophila, Vibrio parahaemolyticus*, and *Campylobacter jejuni*. To overcome the drawbacks of the Gram-stain and culture techniques, we developed a polymerase chain reaction (PCR)–high resolution melt (HRM) assay to detect and identify the species.

*L. pneumophila* is a Gram-negative, non-spore forming, aerobic bacteria that naturally occurs in freshwater lakes, rivers, rainwater puddles, and soils, where it parasitizes amoebas [1,2,3,4,5,6]. It colonizes plumbing systems and cooling towers in man-made structures [1,2,3,4,5]. Heath care buildings, such as hospitals and assisted living communities, tend to have an increased likelihood of colonization by *L. pneumophila* [2]. This tendency to inhabit heating, ventilation, and air-conditioning (HVAC) systems makes for very efficient dispersion of the bacteria, as has been seen in the past with other pathogens such as the H1N1 and SARS viruses [7]. Aerosolized water droplets serve as the primary disease vector; however, inhaled soil particulate can also serve as a less common vector [1,2,3,4,5,6]. Once inhaled, there is a 3-to-10-day incubation period, with the average being 7 days, before the infection symptoms become apparent [4]. Infection with the pathogen causes legionellosis, which is the collective term for the two illnesses *Legionella* species can inflict.

*L. pneumophila* is the strain of *Legionella* responsible for the majority of legionellosis cases in the United States. The species was isolated and identified in 1976 after an outbreak of pneumonia in attendees of the American Legion convention in Philadelphia [2,3,4,5]. The bacteria had colonized the building’s cooling tower, and the air conditioning system then carried the pathogen throughout the building. The illness caused by *L. pneumophila* was subsequently termed Legionnaire’s Disease; it consists of pneumonia, which can be accompanied by flu-like symptoms, nausea, diarrhea, and confusion. Individuals at risk of infection include the elderly, immunocompromised, smokers, and individuals with other underlying health conditions. This at-risk demographic is already liable to struggle with recovering from a severe case of pneumonia, and their odds of survival are not improved when *L. pneumophila* is the causative agent. As a Gram-negative bacterium, *L. pneumophila* release endotoxins that induce endotoxic shock if the bacteria manage to spread to the bloodstream and cause septicemia [8]. The Center for Disease Control (CDC) lists the overall average death rate from Legionnaire’s Disease as 1 in 10; however, if the infection begins while the host is already in a healthcare facility prior to infection then the death rate jumps to 1 in 4 cases [9]. Fortunately, Legionnaire’s Disease rarely manages person-to-person transmission due to requiring the inhalation of an infectious dose of the pathogen in aerosolized droplets, usually produced by coughing, by a secondary at-risk individual [10]. The rarity of this occurrence in relation to an outbreak of legionellosis keeps this pathogen to a low native pandemic potential [6].

There is a less severe permutation of legionellosis, referred to as Pontiac Fever, which forgoes the pneumonia associated with Legionnaire’s Disease. Pontiac Fever claimed its name in 1968 due to an outbreak among individuals who spent time in the Health Department of Pontiac, Michigan [2]. It lacks the added severity caused by the pneumonia that characterizes Legionnaire’s Disease and generally runs its course within a week.

The CDC reports that the incidence of legionellosis has been steadily increasing since 2000, with the highest reported incidence occurring in 2018 and a sharp dip in 2019, which may be due to lessened reporting during the COVID-19 pandemic as opposed to reduced cases as the cases rebounded in 2021 [9]. Legionellosis is a notifiable disease. The vast majority, around 98–99%, of reported legionellosis cases in the United States are Legionnaire’s Disease as a result of a *L. pneumophila* infection, and the remainder are either Pontiac Fever or extrapulmonary legionellosis [9,11,12,13]. Extrapulmonary legionellosis is an *L. pneumophila* infection of a site other than the lungs such as an infection occurring at a surgical site or myocarditis.

The CDC has been providing surveillance reports of the incidence and outcome of *L. pneumophila* infections since 2014. There was no notable decrease in the fatality rate for six years’ worth of reporting, with the death rate averaging 6.6% [11,12,13], indicating either no improvement in the treatment regimen or a lack of proper identification in a timely enough manner for proper treatment to be effective. Early detection can lead to better treatment outcomes for patients.

Other waterborne pathogens present their own forms of threat to human health and may have reason to be tested for along with *L. pneumophila* when inspecting water quality.

*C. jejuni* is a Gram-negative, non-spore forming, microaerophilic species that is a natural component of the microbiome of the gastrointestinal tracts of many bird species as well as cattle. Human infections with *C. jejuni* are caused by ingesting contaminated food or water, and the resulting infection is termed campylobacteriosis [14]. Campylobacteriosis symptoms include bloody diarrhea, fever, nausea, vomiting, and stomach cramps and usually last around a week, with most cases not requiring medical intervention. Individuals who are elderly, pregnant, or immunocompromised are at risk for more severe symptoms or septicemia and may require medical intervention. An oral antibiotic regimen is typically all that is required. Some individuals may become infected with an antibiotic-resistant strain of *C. jejuni* that is capable of surviving prolonged exposure to one of the antibiotics most commonly used to treat it [14]. A bout of campylobacteriosis may also inflict a lasting reminder of the experience in the form of complications including temporary paralysis, irritable bowel syndrome, reactive arthritis, or Guillain–Barré Syndrome (GBS). Around 1 in 1000 cases of campylobacteriosis result in GBS that causes muscle weakness, tingling sensations, and paralysis [14]. Recovery can be a years-long process and permanent nerve damage may remain. In 2022, in the United States the majority of reported *C. jejuni* infections were foodborne rather than waterborne and responsible for the greatest number of diarrheal illnesses overall [14,15]. Other *Campylobacter* species contribute to the CDC’s surveillance data, but these other species only account for approximately 10% of Campylobacteriosis in the United States [14]. In 2022, *Campylobacter* species were responsible for nearly 10,000 infections, 20% of which required hospitalization and <1% of which were fatal [15].

*V. parahaemolyticus* is a Gram-negative, non-spore forming, facultative anaerobe that occurs naturally in salt and brackish water, including in the Chesapeake Bay in Maryland, USA [16]. Illness caused by *V. parahaemolyticus* is acquired either through ingesting contaminated seafood or water or through exposing an open wound to contaminated water. Vibriosis, the intestinal illness caused by *V. parahaemolyticus*, is characterized by watery diarrhea, nausea, vomiting, and fever. Symptom onset is typically within 24 h of ingestion and abates within three days. Individuals who are at greater risk of infection by *V. parahaemolyticus* include those who have recently undergone stomach surgery, those who regularly take antacids, and immunocompromised individuals. Fatalities are usually caused by the pathogen escaping the gut and causing septicemia and endotoxic shock. According to the CDC, the majority of *V. parahaemolyticus* infections are caused by eating contaminated seafood [16,17]. Of the 655 cases of vibriosis reported in 2019, 20% required hospitalization and 1% resulted in death [17].

It should be noted that clinical identification of the species responsible for foodborne illnesses is not frequently performed, so cases and hospitalizations caused by both *C. jejuni* and *V. parahaemolyticus* are underreported. When identifying the bacterial cause of an illness, the most widely used method is culturing the bacteria. A sample is taken from the affected individual. The sample is cultured in a petri dish at 37 °C and the microbes present that grow under the conditions are detected. Individual colonies are selected and transferred to a separate growth plate for further growth and identification. Morphological features, stains, and exposure to different types of growth media are used to determine the identity of the bacteria. The whole process generally takes 3 to 5 days for most bacterial pathogens. This is a reliable and inexpensive method, but the time required between sample acquisition and species identification may be more than a dangerously ill individual can withstand. This issue of time is exacerbated when the infectious species is difficult to grow in culture, such as some strains of *Legionella* that exist in a viable but non-culturable state [1,5]. There are alternative tests for legionellosis, such as a urine antigen test and serological tests, but these also have their limitations. The most frequently used urine antigen test for *Legionella* is only capable of identifying *L. pneumophila* serotype I, which is too specific if another serotype is the cause, and the other urine antigen test is less reliable [4,5]. Serology is neither particularly accurate nor specific and takes, at minimum, 3 weeks [4].

An ELISA assay for *L. pneumophila*, usually either IgM or IgG + IgM, has a sensitivity between 30 and 92%, depending on the duration of the infection, a specificity of 97–100%, and will take anywhere from 90 min to 3 h [18,19,20]. Immunofluorescent assays (IFA) use IgM, IgG, IgA, or polyimmunoglobulin and have a lower sensitivity than ELISA tests and a comparable specificity [18,19,20]. Both ELISA and IFA use blood serum for the testing. The antigenuria urine tests for *L. pneumophila* have a sensitivity of between 50 and 70% and higher specificity than ELISA [18,21,22]. While these assays are validated for clinical testing, they are only useful for specific serotypes of *L. pneumophila* based on which antibodies they test for. The majority of clinical cases are identified as serotype 1, which is the serotype the urine antigen test is optimized for. Additionally, none of these tests are useful for the identification of *L. pneumophila* in an environmental sample such as those that would be collected while investigating the source of an outbreak. An *L. pneumophila* PCR assay was created in 2011 and is both highly accurate and sensitive, but only targets one serotype out of the 15 identified *L. pneumophila* serotypes [23]. This assay can be utilized in both clinical and environmental samples; however, the extreme specificity for a single serotype will prevent detection of the 14 other strains.

If an acceptable assay was created, then real-time PCR testing with the proper primers could take as little as 1 h and would have both high sensitivity and high specificity to the species, which would make it the best option for identification in patients who can produce a sputum sample [4].

The purpose of this study was to design and test primers that will allow for rapid PCR melt identification of the waterborne pathogen *L. pneumophila*, along with other Gram-negative waterborne pathogens. *L. pneumophila* is an active threat to human health. Primers that meet sensitivity and specificity standards can be added to a mix of primers that has already been created to identify various potential infectious agents. This multiplex could then rapidly identify the species behind an infection, allowing a more targeted investigation into the cause of an outbreak and better tailoring of clinical treatment.

## 2. Materials and Methods

### 2.1. Bacterial Strains

There were twenty-two bacterial strains used in the course of this research. Sixteen of the bacterial strain standards were purchased from the American Type Culture Collection (ATCC, Manassas, VA, USA), and the remaining six were sourced from the Towson University Biology department, Midwest Culture Service, Carolina Biological (Burlington, NC, USA) and Ward’s Natural Science (Rochester, NY, USA) (Table 1). The bacterial DNA was delivered as extracted and lyophilized DNA, which was then reconstituted in 100 μL of nuclease-free water. A 5 μM stock solution of each bacterial DNA was created and 1 ng/µL dilution was made of each strain for use in the specificity testing. To calculate the volume of concentrated DNA needed to create the 5 μM stocks, the original concentration was obtained using a ThermoScientific NanoDrop 2000. Serial dilutions to 0.001 ng/µL were also created with the *L. pneumophila*, *V. parahaemolyticus*, and *C. jejuni* DNA to test the primers’ sensitivity.

### 2.2. PCR Primers

The oligonucleotide primers were designed using published gene sequences available from the National Center for Biotechnology Information (NCBI, https://www.ncbi.nlm.nih.gov/nucleotide/, accessed on 13 June 2024). The gene selected for *L. pneumophila* is the macrophage infectivity potentiator (*mip*) gene (Table 2), which is a key virulence factor that inhibits macrophage phagocytosis, which lessens the immune response to a legionellosis infection [24]. The IDT OligoAnalyzer Tool v.3.1 was used to design appropriate primers for both the newly designed and the previously published primers that were chosen as multiplex partners. Primer specificity was checked against the NCBI database using the Basic and Local Alignment Search Tool nucleotide (BLASTn) and the site’s default settings. Amplicon size was intentionally varied between the different species’ primer sets to enable the use of gel electrophoresis as a second source of identity verification. The designed primers were purchased from IDT (Coralville, IA, USA). The primers’ concentrations were quantitated using a NanoDrop 2000 (ThermoFisher Scientific, Frederick, MD, USA). The IDT primer stocks were then used to create diluted stocks with concentrations of 5 µM.

### 2.3. PCR Reaction Conditions and HRM Analysis

The PCR–HRM assays were performed on a Rotor-Gene Q real-time PCR instrument (Qiagen, Hilden, Germany). PCR–HRM analysis was introduced by Sanford and Wittwer in 2013 and applied to species differentiation by Elkins et al. in 2016 [25,26]. The assays were performed using pre-amplification touchdown and fluorescence detection at the HRM step. The run parameters are as follows: the initial hold at 95 °C for 10 min, a second hold at 72 °C for 5 min, a third hold at 45 °C for 1 min, 40 cycles with a denaturation at 95 °C for 15 s, primer annealing for 15 s at 60 °C, and extension for 15 s at 72 °C with acquiring in the green channel and touchdown of the annealing temperature, which lasts for 10 cycles and decreases the annealing temperature by 0.5 °C per cycle, and finally the HRM ramped from 65 °C to 95 °C with an increase of 0.3 °C every 3 s and gain optimization turned on.

The basic non-multiplexed sample assay was prepared in 20 µL PCR tubes that contained 8 µL of 2.5× LightScanner master mix (BioFire Defense, Murray, UT, USA), 1 μL of 5 µM forward primer, 1 μL of 5 µM reverse primer, 9 μL of nuclease-free sterile water, and 1 μL of 1 ng/μL DNA. A no-template DNA control was run with each assay, which contained the same constituents but replaced the 1 μL of 1 ng/μL target DNA with an additional 1 μL of nuclease-free sterile water. Each assay was performed in triplicate to ensure the results were consistent.

The basic sensitivity testing consisted of making a serial dilution of the target DNA standard. Two serial dilutions were created from 1 ng/µL stock solutions; the first was a dilution series that ran from 0.1 ng/µL to 0.001 ng/μL and the second dilution series was prepared from 0.5 ng/µL to 0.005 ng/μL. The assays were then prepared as described above and run at least three times.

The basic specificity testing consisted of testing the primers against each of the bacterial species included in Table 1. Each assay’s specificity was tested in triplicate.

The *L. pneumophila* and *V. parahaemolyticus* duplex assay used a 1:1 primer ratio, which then required a slight alteration to the quantity of water used in the assay setup, bringing the amount used down to 7 μL for samples and 8 μL for the no-template controls. The duplex assay was subjected to both sensitivity and specificity testing to determine its efficacy.

The *L. pneumophila, V. parahaemolyticus*, and *C. jejuni* triplex assay used a mix of the primers in a 1:1:1 ratio. The amount of water used in the assay was brought down to 3 µL for the three samples and 6 µL for the no-template controls. The triplex assay was subjected to sensitivity and specificity testing.

Graphs were prepared in TeeChart Office software (version 2.0). The melt curve is presented as the positive peak of derivative of the fluorescence decrease.

### 2.4. Sizing Using Agarose Gel Electrophoresis

Gel electrophoresis was used to verify the size of the amplified DNA fragments. A 3% agarose gel was prepared with 0.75 g agarose powder and 25 mL 1× TAE. The samples were heated at 95 °C for 2 min and snap cooled for 5 min on ice in preparation for electrophoresis in order to ensure that the DNA was single-stranded. Each well was then loaded with either 5 μL of sample or 5 μL of the Fast Ruler Ultra-Low Range DNA Ladder (200, 100, 50, 10 bp) and TrackIt 100 bp ladder (2000, 1500, 1200, 1000, 900, 800, 700, 600, 500, 400, 200, 100 bp; both ladders are from Thermo Fisher Scientific, Frederick, MD, USA), 1 μL of 6× Orange loading dye, and 1 μL of 50× SYBR green dye. The gel was run on a Horizon 58 for 50 min at 120 volts. After the electrophoresis was completed, the gel was placed on a UV transilluminator and documented. The bands were measured, and the log of the base pair amplicon length was plotted against the distance travelled. A best fit line and line equation was used to compute the size of the amplicons.

## 3. Results

### 3.1. Repeatability Testing for the Singleplex and Multiplex Assays

**Figure 1:**
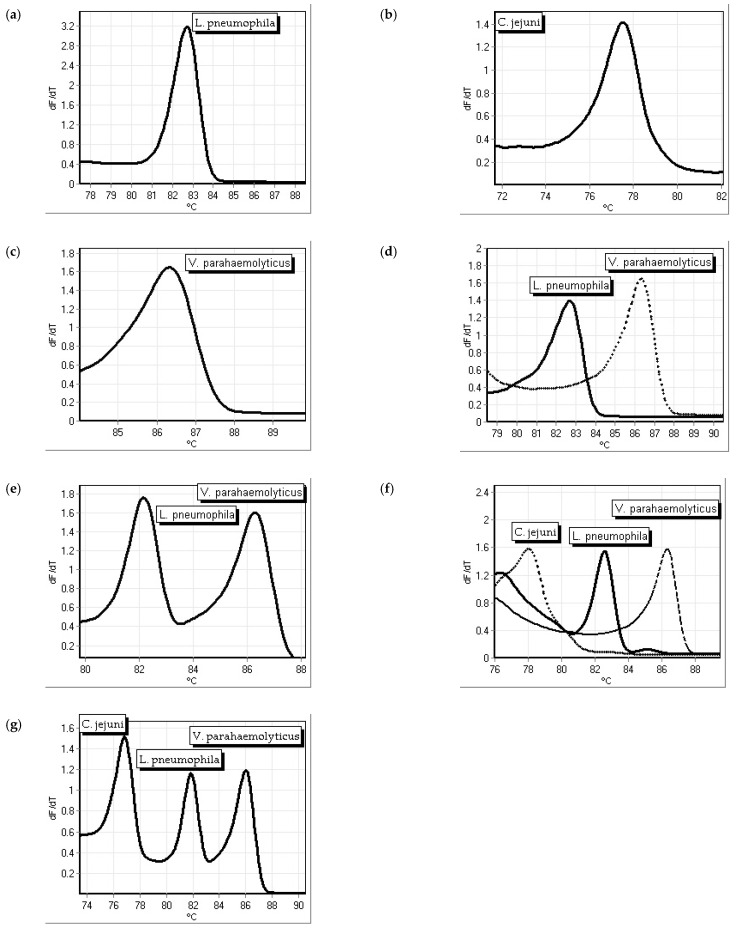
PCR melt curves of (**a**) *L. pneumophila mip*, (**b**) *C. jejuni cadF*, and (**c**) *V. parahaemolyticus thl* primers as single primer assays. Melt curves of *L. pneumophila mip* primers as a duplex assay with *V. parahaemolyticus thl* primers (**d**) testing bacterial DNA individually as a duplex assay and (**e**) testing a bacterial DNA mixture as a duplex assay. Melt curves of *L. pneumophila mip* primers as a triplex assay with *V. parahaemolyticus thl* and *C. jejuni cadF* primers (**f**) testing bacterial DNA individually as a triplex assay and (**g**) testing a bacterial DNA mixture as a triplex assay.

**Figure 2:**
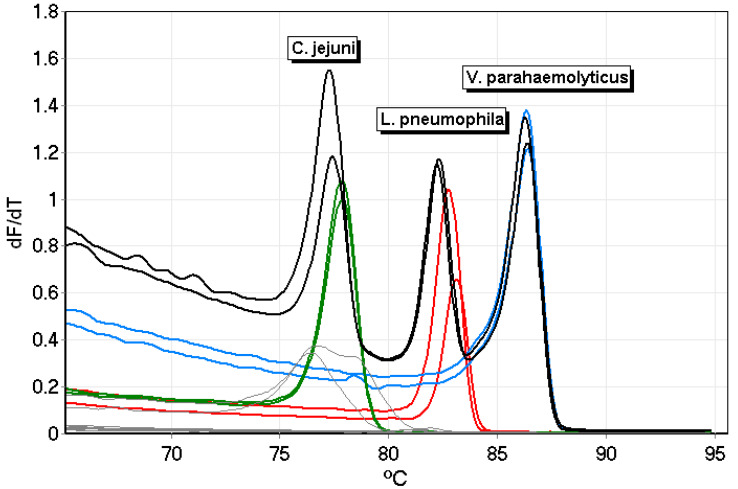
PCR–HRM results from repeatability testing of the primers sets for *C. jejuni* (green line), *L. pneumophila* (red line), and *V. parahaemolyticus* (blue line) and the triplex assay (solid black line). The negative amplification controls are shown with the gray lines (primers and master mix). The initial hold was 95 °C for 10 min followed by 40 cycles of 15 s each at 60 °C, 72 °C, and 95 °C, including the first ten cycles of touchdown from 60 °C to 55 °C in 0.5 °C increments, hold at 72 °C for 5 min, hold at 45 °C for 60 s, and melt from 65 °C to 95 °C, increasing by 0.3 °C every 3 s with gain optimization and detecting HRM.

The *L. pneumophila mip* primer set was tested numerous times and displayed a melting temperature of 82.84 ± 0.19 °C (*n* = 46) (Figure 1a). While the melt temperature for the *L. pneumophila* primer set was 82.84 ± 0.19 °C (Figure 1a), on average, it was 78.10 ± 0.58 °C for *C. jejuni* (Figure 1b), and 86.74 ± 0.65 °C for *V. parahaemolyticus* (Figure 1c). The melt temperatures were retained when the primers were tested as a duplex (Figure 1d,e) and triplex (Figure 1f,g) and each bacterium was detected and identified. Repeatability was tested several times; samples, in duplicate from another run, are shown in Figure 2. *C. jejuni* is presented with the green line, *L. pneumophila* is presented with the red line, and *V. parahaemolyticus* is presented with the blue line. The triplex assay is presented with the solid black line and the no-template controls are shown in gray.

### 3.2. Confirmation of the Amplicons

**Figure 3:**
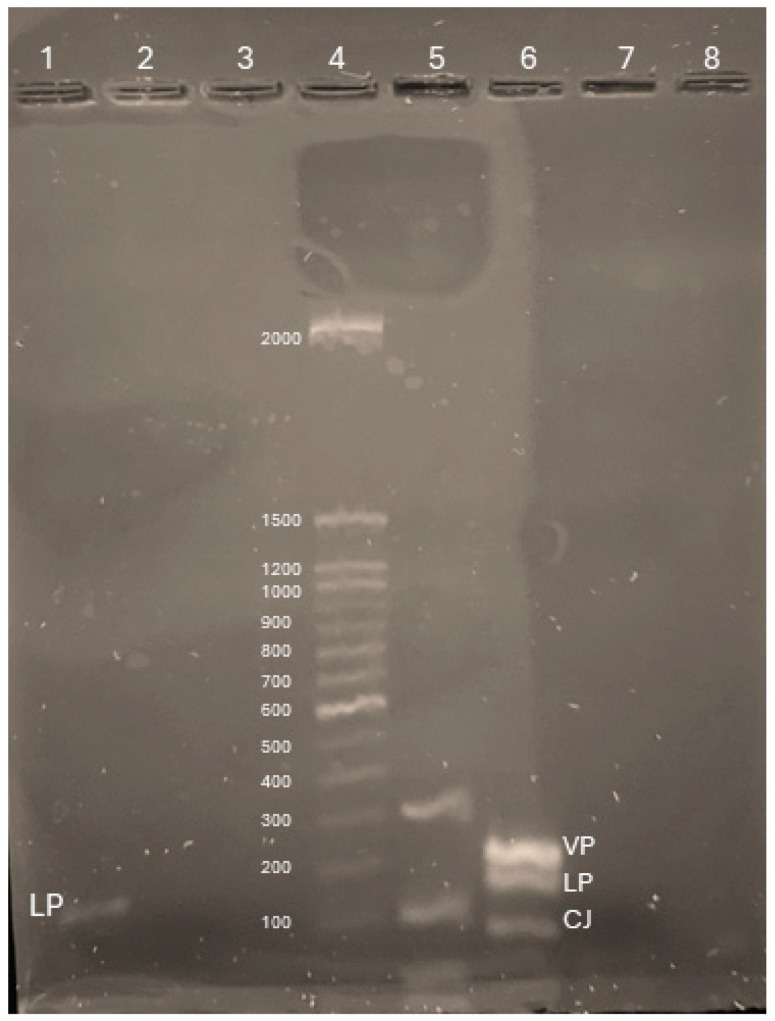
Results from 3% agarose gel with primer sets for *L. pneumophila* (LP), *V. parahaemolyticus* (VP), and *C. jejuni* (CJ) and target DNA each (lanes 1, 2, and 3, respectively), ThermoFisher TrackIt 100 bp DNA ladder (sizes labelled) (lane 4), Fast Ruler Ultra-Low Range DNA ladder (lane 5), triplex primer set with all three target DNAs (lane 6, species labelled), and the negative ampflification control with the triplex primers was run in lane 7.

Gel electrophoresis was performed to check that the intended DNA targets were being amplified, and the expected fragments were identified using the samples from Figure 2. The amplicons for the *L. pneumophila mip* primers (lane 1) and species amplified by the triplex (lane 6) are of the expected relative and predicted sizes (Figure 3) according to the TrackIt 100 ladder. The triplex primers produced a small primer dimer peak, but a band was not visible (lane 7). The ultra-low molecular weight ruler top band did not run as expected, potentially due to the addition of the SYBR in the ladder.

### 3.3. Sensitivity Testing

**Figure 4:**
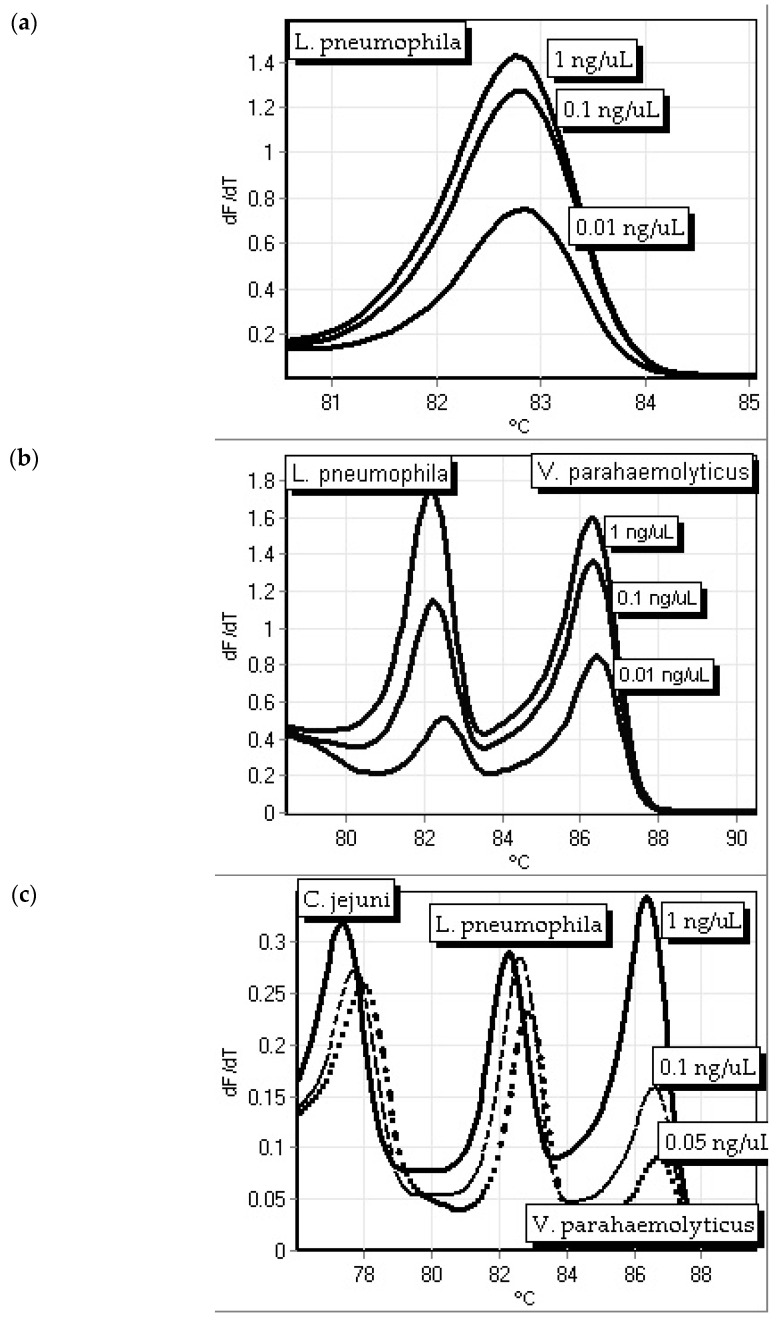
Sensitivity of *L. pneumophila mip* primers as a (**a**) single primer assay, (**b**) duplex assay with *V. parahaemolyticus thl* primers (left peak) testing a mixture of *L. pneumophila* and *V. parahaemolyticus* DNA with concentrations as shown for both, and (**c**) triplex assay with *V. parahaemolyticus thl* and *C. jejuni cadF* primers testing a mixture of *L. pneumophila*, *V. parahaemolyticus*, and *C. jejuni* DNA in triplicate (1 ng/µL, solid black line; 0.1 ng/µL, dashed line; 0.05 ng/µL, dotted line).

The sensitivity of the primers was tested. The sensitivity of the *mip* primer set in a single primer assay are shown down to 0.01 ng/µL for the *L. pneumophila* singleplex (Figure 4a), down to 0.01 ng/µL in a duplex with *V. parahaemolyticus tlh* primers (Figure 4b), and down to 0.05 ng/µL in triplex with *V. parahaemolyticus* and *C. jejuni* (Figure 4c). When in the duplex assay, the *L. pneumophila* primers’ reliable sensitivity decreased to 0.01 ng/µL, while the *V. parahaemolyticus* primers’ sensitivity remained reliable out to 0.01 ng/µL. The triplexed assay consisted of *L. pneumophila mip* primers, *V. parahaemolyticus tlh* primers, and *C. jejuni cadF* primers. The *L. pneumophila* primers were reliable down to 0.005 ng/µL, *V. parahaemolyticus* primers dropped to 0.05 ng/µL, and the *C. jejuni* primers were reliable to 0.05 ng/µL.

### 3.4. Specificity Testing

**Figure 5:**
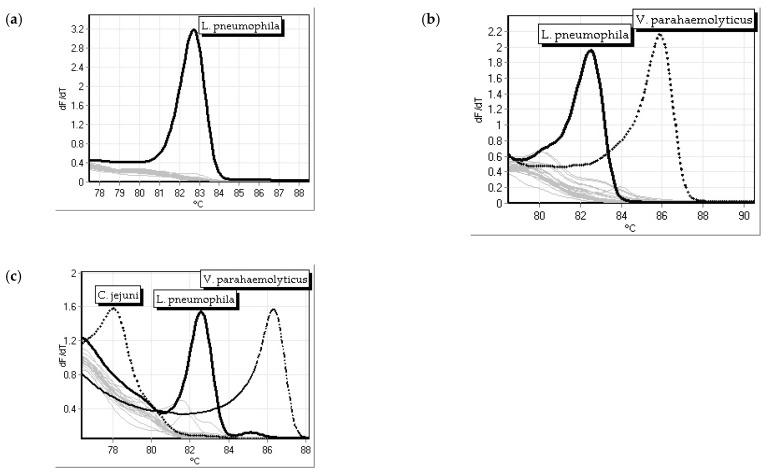
Specificity of *L. pneumophila mip* primers tested against 21 other bacterial strains (gray lines) as a (**a**) single primer assay (solid black trace), (**b**) duplex assay with *V. parahaemolyticus thl* primers (dot black trace), and (**c**) triplex assay with *V. parahaemolyticus thl* (dash dotted black trace) and *C. jejuni cadF* (dot black trace) primers.

In specificity testing, the *mip* primers proved to be specific to *L. pneumophila* in single primer assays, the duplexed assay, and the triplexed assay (Figure 5). The specificity of *L. pneumophila* (dark black trace) using *mip* primers was tested against 21 other bacterial strains (Table 1), shown with light gray lines in a single primer assay (Figure 5a), duplex assay with *V. parahaemolyticus thl* primers (Figure 5b), and a triplex assay with *V. parahaemolyticus thl and C. jejuni cadF* primers (Figure 5c). The *mip* primers amplified the *L. pneumophila* bacterial DNA (dark black trace) but not the other species (light gray lines). The *C. jejuni cadF* primers amplified the *C. jejuni* bacterial DNA (dotted line), and the *V. parahaemolyticus thl* primers amplified the *V. parahaemolyticus* bacterial DNA (dash dot line); neither primer set amplified the twenty-one other species in the panel (light gray lines) from Table 1.

## 4. Discussion

The PCR primer set developed for *L. pneumophila* was developed to be specific to the bacterium by targeting the *mip* gene, which acts as an indicator for the *Legionella* species in addition to encoding a key virulence factor [24]. Validation testing indicates that the primer set has a unique melt temperature that is reliable and repeatable. Paired with the confirmation of proper amplicon size and identity, provided by gel electrophoresis, this displays that the *mip* primer set is accurately amplifying the target DNA.

The single primer assay was tested for specificity against 21 other, non-*Legionella*, bacterial strains. The *mip* primers did not produce a melt curve, and therefore did not amplify any DNA other than the target species. Specificity in the single primer assay is required before multiplexing is considered. To achieve an acceptable degree of specificity, the primer set must only amplify the target bacterial DNA. Sensitivity testing of this single primer assay utilized a dilution series with *L. pneumophila* DNA to produce a set of consistently decreasing DNA concentrations. The *L. pneumophila* primers produced reliable melt curves, distinguishable from instrumental background noise, with concentrations as low as 0.01-0.005 ng/µL of DNA depending on the primer combinations. The 2011 Mérault et al. assay estimated their water assay to have sensitivity comparable to the iQcheck Quanti *Legionella* kit, which operates in genomic units (GU) and has a quantification limit of 480 GU/L [22]. The sensitivity is similar to Reuter et al.’s 2020 *L. pneumophila* melt assay using the 16S gene [27]. They reported the sensitivity to 0.002 ng/µL, but the peak was similar in size to the background and to ours; we called the sensitivity at the more conservative, reproducible, and well above background 0.005 ng/µL in the triplex.

As a single primer assay, the *L. pneumophila mip* primers’ primary utilities are in the clinical identification of this disease-causing pathogen and the forensic investigation as to the source of a Legionnaire’s Disease outbreak using swabs. As buildings that were seeing little use during the COVID-19 outbreak are experiencing an upswing in use, there is likely to be an increase in *L. pneumophila* infections; HVAC and other water sources, which were rarely disturbed while the lockdowns were in effect, will have made ideal habitats for this pathogen to proliferate [5].

The primers for *V. parahaemolyticus* targeted the *tlh* gene (Table 2). The *V. parahaemolyticus* thermolabile hemolysin (*tlh)* gene codes for an enzyme that lyses human erythrocytes and serves as a species marker for this species of bacterium [28,29], and the primers’ melting point falls at 86.74 ± 0.65 °C [18]. Following a successful demonstration as a single primer assay, the *mip* primers were multiplexed with first *V. parahaemolyticus thl* primers, chosen from primer sets previously studied [18] and published by the lab. *V. parahaemolyticus* was selected as a multiplex partner due in part to it’s nature as a waterborne pathogen, and in part due to the fact that it’s melt curve is different enough from the *L. pneumophila* melt curve that it would be readily differentiable. Previously, the primers were shown to be sensitive at 1 ng and nearly baseline alone at 0.01 ng/µL [18], but, in the triplex in this study, they were sensitive to 0.05 ng/µL.

This duplex assay underwent specificity and sensitivity testing in the same manner as the single primer assay. The duplex only amplified the two target DNAs and did so in a manner that produced two easily distinguished melt curves when both target DNAs were present. Sensitivity testing found a ten-fold reduction in sensitivity for the *L. pneumophila* primers when in the duplex. This combination is still a successful duplex assay. The current clinical identification methods have sensitivities as poor as 30–50% and only reliably detect one serotype of *L. pneumophila* [18,19,20,21].

Considering the success of the duplex, another set of primers previously published by the lab was added to produce a triplex [30]. The selected species, *C. jejuni*, was mostly selected due to its melt curve temperature being lower than both that of the *L. pneumophila* and the *V. parahaemolyticus* primers. Several other species were attempted before *C. jejuni* was selected, but all had melt curves that fell too close to the established duplex. The primers for *C. jejuni* targeted *cadF* (Table 2). The *Campylobacter* adhesion to fibronectin (*cadF*) gene codes for an outer membrane fibronectin-binding protein, which assists the pathogen in binding to host cells [31]. Sensitivity and specificity testing were once more tested. Once again, only the three target DNAs were amplified and the three melt curves were distinct. Interestingly, the *L. pneumophila* primers halved their sensitivity when triplexed as compared to the single primer assay, and the overall amplification of all three pathogens dropped significantly when DNA from all three pathogens was present in a sample. The sensitivity of the *C. jejuni cadF* primers was reported to be 0.0005 ng/µL [30] and reduced a hundred-fold in the multiplex. The melt curve shifted slightly in maximum temperature as the amplifiable DNA concentration decreased; this is a behavior displayed by other primer sets in similar circumstances [18,30]. PCR assays, especially multiplexes, can be especially useful in the early detection and identification of pathogens without culturing.

## 5. Conclusions

The *L. pneumophila mip* primer set is fully functional as a single primer PCR–HRM assay, with results in less than three hours. A hospital could use a PCR assay and this primer set to identify if *L. pneumophila* bacteria is the cause of an infection and confirm by sequencing, if needed. The current pathogens detected by the multiplexes with the *L. pneumophila* primers have not yet been detected in a multiplex. However, from a clinical perspective, which is likely where a multiplex would be most useful, the preferred multiplex partners for future assay development should be pathogenic bacteria that also cause pneumonia symptoms and are thus more difficult to differentiate from *L. pneumophila*. Rapid detection and identification is often essential for appropriate treatment.

*L. pneumophila* particularly impacts a subset of the population already vulnerable to respiratory infections, such as the elderly, the immunocompromised, or those who are already ill. *L. pneumophila* colonizes HVAC systems, which can easily spread pathogens throughout a building [1,7], so a non-functional filtration system with a functional blower would create a significant dispersal system affecting an entire building or nursing facility. An assay such as the one demonstrated in this work would be essential to the rapid detection and response in clinical cases.

Further research is required before the *L. pneumophila* PCR assay will be ready for clinical use. Prior to being used, the *L. pneumophila* PCR assay’s amplified DNA product needs to be sequenced so the identity of the PCR product can be conclusively verified. The assay needs to be run against human DNA to ensure there will be no cross-amplification, and other master mixes need to be tested with the assay. Testing with clinical samples through collaboration or an independent evaluation will need to be conducted prior to validation and implementation.

## Figures and Tables

**Table 1:** Bacterial DNA utilized for specificity testing.

Bacterial Strain	Source
*Acinetobacter baumanii*	ATCC
*Bacillus cereus*, str. NRRL B-568	ATCC (10876D-5)
*Bacillus megaterium*	TU Biology collection
*Bacillus subtilis*, str. 168	ATCC (23857D-5)
*Bacillus thuringiensis*, str. USDA H522	ATCC (35646D-5)
*Campylobacter jejuni*, subsp. Jejuni	ATCC (33560D-5)
*Citrobacter freundii*	Carolina Biological
*Clostridium difficile*, str. 90556-M6S	ATCC (9689D-5)
*Enterobacter aerogenes*	TU Biology
*Escherichia coli*, str. MG1655	ATCC (700926D-5)
*Escherichia coli,* str. FDA strain Seattle 1946	ATCC (25922)
*Klebsiella oxytoca*, str. MsA1	ATCC
*Listeria monocytogenes*, str. EGDe	ATCC (BAA-679D-5)
*Legionella pneumophila,* str. Philadelphia-1	ATCC (33152D-5)
*Micrococcus luteus*	Midwest Culture Service
*Pseudomonas aeruginosa,* str. *PAO1-LAC*	ATCC (47085D-5)
*Salmonella enterica*, subsp. Enterica	ATCC (700720)
*Serratia marcescens*	Carolina Biological
*Shigella flexneri* type 2, str. 24570	ATCC (29903D-5)
*Staphylococcus capitis* subsp. *capitis* Kloos and Schleifer	ATCC (35661)
*Staphylococcus saprophyticus*	Ward’s Natural Science
*Vibrio parahaemolyticus*, str. EB101	ATCC (17802D-5)

**Table 2:** Gene target and PCR primers designed and tested for *L. pneumophila* (LP) and gene target and PCR primers for multiplex partners *V. parahaemolyticus* (VP) and *C. jejuni* (CJ).

Species Abbrev.	Gene Target	Forward Primer (5′ to 3′)	Reverse Primer (5′ to 3′)	Amplicon Size (bp)
CJ	*cadF*	TGCTATTAAAGGTATTGATGTAGGTGA	CAGCATTTGAAAAATCCTCAT	83
LP	*mip*	AGATTTGATGGCTAAGCGTACT	AACCACTTGGCAATACAACA	122
VP	*tlh*	ACTGGATTTCGCTTTGCCCTCAATGA	GTTCTGAGTTCGATAACCTCTTGTGTGGATTAAG	146

## Data Availability

The original contributions presented in the study are included in the article; further inquiries can be directed to the corresponding authors.
